# Global DNA hypomethylation in leukocytes associated with glioma risk

**DOI:** 10.18632/oncotarget.18739

**Published:** 2017-06-28

**Authors:** Jie Shen, Renduo Song, Ye Gong, Hua Zhao

**Affiliations:** ^1^ Department of Epidemiology, The University of Texas MD Anderson Cancer Center, Houston, TX, USA; ^2^ Department of Neurosurgery, Huashan Hospital, Shanghai Medical College, Fudan University, Shanghai, China

**Keywords:** glioma, global DNA hypomethylation, leukocytes, cancer risk

## Abstract

Global DNA hypomethylation in leukocytes has been associated with increased risk for a variety of cancers. However, the role of leukocyte global DNA hypomethylation in glioma development, if any, is largely unknown. To define this role, we performed a case-control study with 390 glioma patients and 390 controls with no known cancer. Levels of 5-methylcytosine (5-mC%), a marker for global DNA methylation, were measured in leukocyte DNA. Overall, median levels of 5-mC% were significantly lower in glioma cases than in controls (3.45 vs 3.82, P=0.001). Levels of 5-mC% differed significantly by age and sex among controls and by tumor subtype and grade among glioma cases. In multivariate analysis, lower levels of 5-mC% were associated with a 1.31-fold increased risk of glioma (odds ratio = 1.31, 95% confidence interval = 1.10-1.41). A significant dose-response trend was observed in quartile analysis (P=0.001). In an analysis further stratified by clinical characteristics at baseline, the association between lower levels of 5-mC% and glioma risk was evident only among younger participants (age <52 years), women, and those with aggressive tumor characteristics, such as glioblastoma subtype, high tumor grade (grade III or IV), and absence of *IDH1* mutation. Our findings indicate that global DNA hypomethylation in leukocytes may contribute to the development of glioma and that the association is affected by age, sex, and tumor aggressiveness.

## INTRODUCTION

Development of human gliomas involves a complex combination of cancer-predisposing systemic genetic alterations in association with known or unknown environmental risk factors and somatic genetic alterations that ultimately drive the glial cells to abnormal proliferation and malignant transformation. Epigenetic alterations, including both global DNA hypomethylation and CpG site–specific hypermethylation, are widely considered key genetic alterations in this process. For example, studies have shown that global DNA hypomethylation in tumor tissues is a common event in the oncogenesis of glioblastoma, the most common and lethal subtype of glioma, and may lead to increased proliferative activity and genomic instability [1]. However, whether global DNA hypomethylation is a systemic genetic event or simply a tissue-specific alteration in the development of glioma is still unclear.

Growing evidence from recent molecular epidemiological studies suggests that the role of global DNA hypomethylation in disrupting genomic integrity and stability and thereby promoting cancer development is a broader, systemic event [2]. For example, global DNA hypomethylation in leukocytes has been linked to elevated risk of at least a dozen different cancers [3–16]. Limited evidence in a few different cancers suggests that levels of global DNA methylation are correlated between targeted tumor tissues and matched leukocytes [17]. Thus, level of global DNA methylation in leukocytes may reveal the general level of genomic stability of an individual, loss of which may predispose to cancer development [12].

While the association between global DNA hypomethylation in leukocytes and cancer risk has been reported in many cancer types, the association with glioma risk has not been explored. To assess this relationship, we carried out a case-control study with 390 glioma cases and 390 controls. Global DNA methylation in leukocytes was measured by quantifying levels of 5-methylcytosine (5-mC%). We also explored whether the association between global DNA hypomethylation in leukocytes and glioma risk could be affected by clinical characteristics at baseline.

## RESULTS

Demographic and clinical characteristics of the glioma cases and controls are shown in Table 1. Among the 390 glioma cases, 125 had low-grade glioma (grade I, n = 9; grade II, n = 116) and 265 had high-grade glioma (grade III, n = 94; grade IV, n = 171). As expected, glioblastoma (43.9%) was the most frequent tumor subtype, followed in frequency by astrocytoma (27.7%) and oligodendroglioma (21.3%). *IDH1* mutation status was available for 263 patients, with 121 positive. 1p/19q co-deletion status was available for 155 patients, with 80 positive.

**Table 1:** Baseline characteristics of glioma case and control participants

Variable	Cases (n=390)	Controls (n=390)	P value
**Age, years, mean (SD)**	52.1 (9.9)	52.4 (10.2)	0.677
**Sex**			
Female	158 (40.5%)	160 (41.0%)	
Male	232 (59.5%)	230 (59.0%)	0.884
**Smoking history**			
Ever smoker	164 (42.1%)	174 (44.6%)	
Never smoker	202 (51.8%)	209 (53.6%)	0.886
Unknown	24 (6.2%)	7 (1.8%)	
**BMI category**			
Normal/underweight	192 (49.2%)	170 (43.6%)	
Overweight	106 (27.2%)	104 (26.7%)	
Obese	92 (23.6%)	116 (29.7%)	0.127
**Tumor subtype**			
Astrocytoma	108 (27.7%)		
Glioblastoma	171 (43.9%)		
Oligoastrocytoma	19 (4.9%)		
Oligodendroglioma	83 (21.3%)		
Pilocytic astrocytoma	9 (2.3%)		
**Tumor grade**			
I	9 (2.3%)		
II	116 (29.0%)		
III	94 (24.1%)		
IV	171 (43.9%)		
***IDH1* mutation status**			
Negative	142 (36.4%)		
Positive	121 (31.0%)		
Unknown	127 (32.6%)		
**1p/19q co-deletion status**			
Negative	75 (19.2%)		
Positive	80 (20.5%)		
Unknown	235 (60.3%)		

SD, standard deviation; BMI, body mass index.

First, we compared the median 5-mC% levels between glioma cases and controls (Table 2). Overall, glioma cases had significantly lower levels of 5-mC% than controls (median, 3.45 vs 3.82, P=0.001). When median 5-mC% levels were compared between the case and control groups according to age group and sex, the case-control difference was significant only among younger study participants (age <52 years; P<0.001) and women (P<0.001), not among older participants or men. When we looked at the influence of smoking status and body mass index (BMI) category, the association remained significant for each subgroup, and the strength of the association was similar between the subgroups. Then, we compared the 5-mC% levels within the case group or the control group by demographic variables. The difference was significant only for age group and sex in the control group. Control participants who were older (≥52 years; P=0.001) and/or men (P=0.026) had lower levels of 5-mC% than their counterparts. In the cases, median 5-mC% levels differed by tumor subtype and grade. Those with a non-glioblastoma tumor had higher median 5-mC% levels than those with glioblastoma (P=0.041). Cases with low-grade glioma had higher median 5-mC% methylation levels than those with high-grade glioma (P=0.039). Those whose tumor was positive for *IDH1* mutation had marginally significantly higher median 5-mC% levels than those whose tumor was negative for *IDH1* mutation (P=0.078).

**Table 2:** Median 5-mC% levels by selected demographic and baseline clinical variables of glioma cases and controls

Variable	Cases (n=390)	Controls (n=390)	P value*
**Overall, median (range)**	3.45 (0.39-19.72)	3.82 (0.87-19.81)	0.001
**Age, years**			
<52	3.49	4.12	<0.001
≥52	3.41	3.49	0.206
P value**	0.116	0.001	
**Sex**			
Male	3.46	3.47	0.472
Female	3.44	3.99	<0.001
P value**	0.758	0.026	
**Smoking status**			
Ever	3.37	3.79	0.003
Never	3.55	3.98	0.005
P value**	0.242	0.329	
**BMI category**			
Normal/underweight	3.46	3.87	0.016
Overweight	3.43	3.73	0.017
Obese	3.43	3.80	0.015
P value**	0.378	0.724	
**Tumor subtype**			
Glioblastoma	3.31		
Non-glioblastoma	3.59		
P value**	0.041		
**Tumor grade**			
I/II	3.61		
III/IV	3.27		
P value**	0.039		
***IDH1* mutation**			
Negative	3.30		
Positive	3.64		
P value**	0.078		
**1p/19q co-deletion**			
Negative	3.43		
Positive	3.47		
P value**	0.652		

*P compares median 5-mC% levels between cases and controls.

**P compares median 5-mC% levels between subgroups defined by selected characteristics.

BMI, body mass index.

Next, we investigated the relationship between 5-mC% level and risk of glioma (Table 3). In the multivariate linear regression analysis with 5-mC% level as a continuous variable, lower 5-mC% levels were associated with a 1.31-fold greater risk of glioma after adjusting for age, sex, smoking status, and BMI (OR = 1.31, 95% CI = 1.10-1.41). When 5-mC% levels were dichotomized into two groups (high or low) using the median 5-mC% level in the control group (3.82), we found that lower levels of 5-mC% were associated with a 1.74-fold increased risk of glioma after adjusting for the covariates (OR = 1.74, 95% CI = 1.30-2.64). In the quartile analysis using 25%, 50%, and 75% values of 5-mC% in the control group as cutoff points, we found that those in the third and fourth (i.e., lower) 5-mC% quartiles had a 1.56-fold or 2.07-fold, respectively, greater risk of glioma than those in the first (i.e., highest) quartile (third, OR = 1.56, 95% CI = 1.02-2.64; fourth, OR = 2.07, 95% CI = 1.44-3.31). A statistically significant dose-response trend was observed across the quartiles (P = 0.001).

**Table 3:** Risk of glioma as estimated by 5-mC% level

5-mC% levels	Cases (%)	Controls (%)	Crude OR (95% CI)	OR (95% CI)*
**Continuous variable**	390 (100%)	390 (100%)	1.36 (1.12-1.39)	1.31 (1.10-1.41)
**Categorical variable**				
By median in controls				
≥3.82	136 (34.9%)	195 (50.0%)	1.00	1.00
<3.82	254 (65.1%)	195 (50.0%)	1.87 (1.39-2.52)	1.74 (1.30-2.64)
By quartile in controls				
1^st^ (highest)	66 (16.9%)	98 (25.1%)	1.00	1.00
2^nd^	72 (18.5%)	97 (24.9%)	1.10 (0.70-1.75)	1.10 (0.63-1.79)
3^rd^	107 (27.4%)	97 (24.9%)	1.64 (1.06-2.54)	1.56 (1.02-2.64)
4^th^ (lowest)	145 (37.2%)	98 (25.1%)	2.20 (1.44-3.36)	2.07 (1.40-3.46)
				P for trend = 0.001

*Adjusted by age, sex, smoking status, and BMI.

OR, odds ratio; CI, confidence interval; BMI, body mass index.

In further stratified analysis, we assessed whether the association between 5-mC% levels and risk of glioma was affected by selected demographic and clinical characteristics. For age group and sex, the association was significant only among younger (<52 years) participants (OR = 2.42, 95% CI = 1.52-4.14) and women (OR = 2.14, 95% CI = 1.32-3.49) but not among older participants or men. When we looked at the tumor characteristics, the association was significant only among those with aggressive tumor phenotypes at baseline, including glioblastoma (OR = 2.24, 95% CI = 1.31-3.92), high-grade glioma (OR = 1.78, 95% CI = 1.19-2.89), and *IDH1* mutation–negative glioma (OR = 1.67, 95% CI = 1.04-3.15).

## DISCUSSION

To our knowledge, our study is the first to investigate the association between global DNA methylation in leukocytes and glioma risk. We found that the levels of 5-mC% were significantly lower in glioma cases than in controls and the association differed by age group and sex. In the risk assessment, lower levels of 5-mC% were associated with increased risk of glioma. In further stratified analysis, we found that the association was significant only among younger participants, women, and those with an aggressive tumor phenotype at baseline.

Our results confirm the positive association between global DNA hypomethylation in leukocytes and cancer risk reported in several other cancer sites, including head and neck [3], testes [18], stomach [4], hepatocellular carcinoma [5], bladder [6–8], colon and rectum [9, 10], breast [12], melanoma [15, 16], and kidney [19]. The consistency among those studies demonstrates that DNA hypomethylation is a key and frequent genetic event contributing to cancer development. It also suggests that the phenomenon of global DNA hypomethylation is likely systemic. Intriguingly, in a recent study of 183 brain tumor patients, Barciszewska et al. observed high correlation of global DNA hypomethylation between tumor tissues and matched leukocytes [17]. They observed similar correlations for breast and colon cancers. Nevertheless, how global DNA hypomethylation increases cancer risk is poorly understood. Although chromosomal instability, reactivation of transposable elements, and loss of imprinting have been proposed as mechanisms underlying the contribution of global DNA hypomethylation to carcinogenesis [20], more research is clearly still needed.

Several studies have reported significant associations between global DNA hypomethylation and the exposure to cancer risk factors [21–24], suggesting that DNA hypomethylation could be the result of carcinogenic exposures. Exposure to ionizing radiation is one of the few known risk factors for glioma. Recent studies in animal models and human subjects have shown that radiation exposure may affect global DNA methylation level. For example, Wang et al. reported that mice exposed to low-dose radiation had lower levels of global DNA methylation in DNA extracted from blood cells than mice not exposed to radiation [25]. In a study of nuclear power plant workers, global DNA methylation levels in DNA from blood samples were lower in radiation-exposed workers than in controls [26]. Further analysis identified a positive correlation between radiation-induced DNA methylation alterations and chromosome aberrations. Because radiation exposure data are not available for our study population, we were unable to assess this relationship in our study.

Consistent with our previous study in melanoma and several other literature reports, our current study found that levels of global DNA methylation in leukocytes were affected by age [16, 27–29] and sex [3, 7, 16, 27–30] in controls. More interestingly, in the stratified analysis, the risk was evident only among younger participants (<52 years) and women, who are historically less affected by glioma. This suggests that the role of global DNA hypomethylation in cancer development is more than a mediator or effector of carcinogen exposure. Similar differences have been reported previously [19]. For example, Mendoza-Perez et al. found that the significant association between global DNA hypomethylation and renal cell carcinoma was limited to younger individuals [19].

Another interesting finding is that 5-mC% levels were significantly lower in high-grade tumors and glioblastoma and marginally lower in *IDH1* mutation–negative tumors than in low-grade tumors, non-glioblastoma gliomas, and *IDH1* mutation–positive tumors. We further observed that the association between 5-mC% level and glioma risk was more evident in the tumors with aggressive phenotypes, that is high-grade tumors, glioblastomas, and tumors negative for *IDH1* mutation. In a previous study in glioma tumor tissues, level of 5-MC% was inversely correlated with the World Health Organization malignancy grade [17], a finding consistent with our results. In another glioma study, Ohka et al. reported that levels of global DNA methylation in tumor tissues were lower in glioblastoma than in low-grade tumors and normal brain tissues [31]. They further reported that level of global DNA methylation was proportional to *MGMT* promoter methylation in gliomas and that higher level of global DNA methylation was a favorable prognostic factor in primary glioblastoma, even compared to *MGMT* promoter methylation. In the current study, *MGMT* promoter methylation data were not available for most of the glioma patients.

A case-control difference in immune cell profiles in leukocytes may partially contribute to the association observed in the current study. A growing body of literature is now defining differentially methylated regions (DMRs) based on immune cellular differentiation. For example, a DMR in *GATA3* is hypomethylated in naïve and memory CD4+ cells compared with CD34+, CD8+, T, and B cells, whereas DMRs in *TCF7* and *Etv5* are hypermethylated in B and T memory cells compared with their naïve counterparts [32]. DMRs in the *FOXP3* locus are methylated in naïve CD4+CD25- T cells, activated CD4+ T cells, and TGF-β–induced adaptive T-regulatory cells, whereas they are completely demethylated in natural T-regulatory cells, which are critical in autoimmune regulation [33]. Unfortunately, immune cell profile data are not available for the cases and controls in our study. Considering that global DNA hypomethylation is likely a systemic effect and the relationship between global DNA hypomethylation and cancer risk is consistent across various types of cancer, the influence of immune cell profiles might be minimal to modest at most. There is no study comparing levels of global DNA methylation levels among immune cell types. However, it would be interesting to investigate in the future whether levels of global DNA methylation differ by immune cell type and how such differences modify the association with cancer risk.

As this is a retrospective case-control study, we cannot establish the temporal relationship and causality between DNA hypomethylation and the risk of glioma development. We can't exclude the possibility of “reverse causation,” although it is improbable. As in all existing studies on global DNA hypomethylation, 5-mC% methylation levels were measured only once in our study. Nonetheless, our data provide evidence supporting a positive relationship between global DNA hypomethylation in leukocytes and glioma risk and suggesting that that the relationship is limited to younger people, women, and patients with an aggressive tumor phenotype. Obviously, additional studies in prospective cohorts are necessary to confirm the observed associations in this study and to further our understanding of the role of global DNA methylation in glioma carcinogenesis.

## MATERIALS AND METHODS

### Study participants

For this case-control study, glioma patients were recruited from The University of Texas MD Anderson Cancer Center (Houston, TX). Detailed information on these cases has been reported previously [34]. All patients with either newly diagnosed or previously treated histopathologically confirmed glioma who were registered at MD Anderson between April 2014 and July 2016 were eligible for our study. Written informed consent was obtained from each study subject. The clinical characteristics for the cases were obtained from medical record review. A total of 390 Caucasian glioma patients were included in the study. Patients were selected at the time of analysis according to the following requirements: 1) newly diagnosed glioma; 2) not receiving a course of chemotherapy or radiation therapy when the blood was drawn; and 3) clinical characteristics at baseline available. Patients with recurrent or progressing glioma were excluded from the study.

IDH1 mutation status was determined by the immunochemistry staining.

Cancer-free controls were drawn from an ongoing melanoma case-control study. They were recruited from unrelated clinic visitors and patient spouses. Written informed consent was obtained from each control participant. The demographic data on the controls were collected from a structured self-administered questionnaire. A total of 390 Caucasian controls were included in the study. The cases and controls were frequency matched on age, sex, and smoking status. The exclusion criteria for both cases and controls included prior cancer diagnosis (except for non-melanoma skin cancer) and any blood product transfusion in the 6 months prior to recruitment. In addition, controls who had been recruited from the Brain Tumor Center were excluded. The study protocol was approved by the Institutional Review Board at MD Anderson.

### Global DNA methylation analysis

Leukocyte DNA was extracted using the QIAamp DNA Blood Maxi Kit (Qiagen, Hilden, Germany). Levels of 5-mC, the marker of global DNA methylation, in the extracted DNA were measured by the 5-mC DNA ELISA Kit (Zymo Research, Irvine, CA) as described previously [16]. This kit uses a unique monoclonal antibody that is both sensitive and specific for 5-mC to detect and quantify DNA methylation. Total DNA methylation level was measured as a percentage of total DNA present in the sample. Briefly, after 100 ng of DNA (20 ng/μL) was bound to the plate at 37°C for 1 hour, the capture and detection anti-5-mC and secondary antibodies were allowed to bind to 5-mC at 37°C for 1 hour, and the relative optical density units were quantified by reading the absorbance at 405 nm using a Synergy microplate reader (BIOTEK, Winooski, VT). The fraction of methylated DNA was proportional to the optical density. Positive and negative controls were included on each plate. A standard curve was generated, and the percentage of 5-mC in a DNA sample (5-mC%) was quantified from the standard curve. The slope of the standard curve was used to calculate the absolute amount of methylated DNA in the DNA sample. The inter-assay coefficient of variation was <10% in the analysis.

### Statistical analysis

Statistical analyses were performed using the STATA software package (version 13, STATA, Inc., College Station, TX). The differences in the distributions of basic demographic variables between cases and controls were evaluated using the chi-squared test for categorical variables and Student *t*-test for continuous variables (Table 1). To examine the differences between cases and controls for the associations between median 5-mC% levels and selected categorical characteristics and the differences between selected categorical characteristics for the median 5-mC% within the case or control group, the Wilcoxon rank-sum test was used (Table 2). Multivariate unconditional logistic regression analysis was used to assess the main effect of 5-mC% level on glioma risk (Table 3). Odds ratios (ORs) and 95% confidence intervals (CI) were calculated. Potential covariates were adjusted in the analysis. 5-mC% levels were examined in several ways, including as a continuous variable, as a categorical variable divided by the median value for the controls, and as a categorical variable based on the quartile distribution for controls. For 5-mC% level as a continuous variable, we compared the results using log-transformed 5-mC% and non-transformed 5-mC%. The strength of the associations was similar and remained statistically significant in both analyses. At here, we only presented the results generated from non-transformed data. Cutoff points for all constructed categorical variables were based on the distribution within the control population. The dose-response was tested for the quartile distribution of 5-mC% level by inserting the mean value of each quartile and then treating the variable as a continuous variable in the logistic regression model. Stratified analysis was performed to assess whether the relationship between 5-mC% levels and glioma risk differed by clinical characteristics at baseline (Table 4).

**Table 4:** Glioma risk estimation by baseline clinical characteristics

Variable	5-mC%	Cases, n (%)	Controls, n (%)	Crude OR (95% CI)	OR (95% CI)*
**Age at diagnosis, years**					
<52	high (≥4.12)	57 (33.5%)	99 (50.8%)	1.00	1.00
	low (<4.12)	140 (66.5%)	96 (49.2%)	**2.53 (1.64-3.93)**	**2.42 (1.52, 4.14)**
≥52	high (≥3.49)	91 (38.5%)	100 (51.3%)	1.00	1.00
	low (<3.49)	102 (61.5%)	95 (48.7%)	1.18 (0.78, 1.79)	1.18 (0.72, 1.96)
**Sex**					
Male	high (≥3.47)	108 (46.6%)	118 (51.2%)	1.00	1.00
	low (<3.47)	124 (53.2%)	112 (48.8%)	1.21 (0.83-1.77)	1.15 (0.64, 2.02)
Female	high (≥3.99)	49 (31.0%)	79 (49.6%)	1.00	1.00
	low (<3.99)	109 (69.0%)	81 (50.4%)	**2.17 (1.34-3.52)**	**2.14 (1.32, 3.49)**
**Tumor subtype**					
Glioblastoma	high (≥3.82)	52 (30.4%)	98 (25.1%)	1.00	1.00
	low (<3. 82)	119 (69.6%)	97 (24.9%)	**2.31 (1.47-3.65)**	**2.24 (1.31, 3.92)**
Non-glioblastoma	high (≥3. 82)	94 (42.9%)	97 (24.9%)	1.00	1.00
	low (<3. 82)	125 (57.1%)	98 (25.1%)	1.32 (0.88-1.98)	1.25 (0.82, 2.19)
**Tumor grade**					
I/II	high (≥3. 82)	57 (45.6%)	98 (25.1%)	1.00	1.00
	low (<3. 82)	68 (54.4%)	97 (24.9%)	1.21 (0.75-1.94)	1.14 (0.64, 2.17)
III/IV	high (≥3. 82)	94 (35.5%)	97 (24.9%)	1.00	1.00
	low (<3. 82)	171 (64.5%)	98 (25.1%)	**1.80 (1.21-2.67)**	**1.78 (1.19, 2.89)**
***IDH1* mutation**					
Negative	high (≥3. 82)	51 (35.9%)	98 (25.1%)	1.00	1.00
	low (<3. 82)	91 (64.1%)	97 (24.9%)	**1.80 (1.13-2.88)**	**1.67 (1.04, 3.15)**
Positive	high (≥3. 82)	53 (43.8%)	97 (24.9%)	1.00	1.00
	low (<3. 82)	69 (56.2%)	98 (25.1%)	1.29 (0.80-2.09)	1.24 (0.70, 2.71)
**1p/19q co-deletion**					
Negative	high (≥3. 82)	32 (42.7%)	98 (25.1%)	1.00	1.00
	low (<3. 82)	43 (57.3%)	97 (24.9%)	1.36 (0.77-2.41)	1.30 (0.65, 2.75)
Positive	high (≥3. 82)	34 (42.5%)	97 (24.9%)	1.00	1.00
	low (<3. 82)	46 (57.5%)	98 (25.1%)	1.34 (0.77-2.35)	1.35 (0.70, 2.81)

*Adjusted by age, sex, smoking status, and BMI; boldface indicates statistical significance.

OR, odds ratio; CI, confidence interval; BMI, body mass index.

## Abbreviations

(IDH): isocitrate dehydrogenase
(BMI): body mass index
